# Supplementary material for: Human papillomavirus (HPV) vaccine uptake and its associated factors among adolescent girls in Kathmandu District, Nepal: A cross-sectional study
Source: PLOS Glob Public Health. 2026 Jan 30;6(1):e0005893. doi: 10.1371/journal.pgph.0005893 (PMC12857967; doi:10.1371/journal.pgph.0005893)
Supplement: S1 File — (DOCX) [file pgph.0005893.s001.docx]

**Data collection tools**

Please fill in the following blanks:

Municipality name:

Ward number:

Participants ID:

Address:

Name of School:

Type of school:

School Address:

Contact no. (Parents):

Distance of school from vaccine delivery site(walking/vehiche): In minute…………

**Provide your answers according to the instructions provided for each set of questions in the following sections.**

| **Section A: Socio- demographic information** | | | |
| --- | --- | --- | --- |
| S.N | Questions | | Your Answer |
| 1. | How old are you? | | ……………………………………. |
| 2. | In which class are you? | | 1. 9 2. 10 |
| 3. | Which ethnicity do you belong to? | | 1. Adibasi/janajati 2. Brahmin/Chettri 3. Newar 4. Dalit 5. Terai/Madhesi 6. Others specify………………. |
| 4. | Which religion do you follow? | | 1. Hinduism 2. Buddhism 3. Christianity 4. Muslim 5. Others…………….specify |
| 5. | What is your mother's educational status? | | Completed class………………… |
| 6. | | What is your father's educational status? | Completed class……………………… |
| 7. | | What is the type of your family? | 0. Nuclear (parents and their own children only)  1. Joint (consisting of grandparents, great-grandparents, paternal grandparents, cousins and their children) |
| 8. | | How many members are there in your family? | ………………………………………… |
| 9 | | Total number of siblings |  |
| 10. | | What is your parent's marital status? | 1. Married 2. Separated 3. Widow 4. Both of them dies |
| 11. | | Which of these media sources do you use the most?  (Select all that apply.) | 1. Newspaper 2. Television 3. Radio 4. Internet 5. None 6. Others specify……………….. |
| 12. | | What is your father's employment status? | 1. Full-time job 2. Part-time job 3. Unemployed |
| 13. | | What is your father's occupation? | 1. Agriculture 2. Building Construction 3. Daily Wages 4. Government Job 5. Private job 6. Business 7. Home-maker(House wife) 8. Foreign Employment 9. I don’t know 10. others specify…………………….. |
| 14. | | What is your mother's employment status? | 1. Full-time job 2. Part-time job 3. Unemployed |
| 15. | | What is your mother's occupation? | 1. Agriculture 2. Building Construction 3. Daily Wages 4. Government Job 5. Private job 6. Business 7. Home-maker(House wife) 8. Foreign Employment 9. I don’t know 10. others specify…………………… |
| 16 | | Who earns money in your family? | 0. Father  1. Mother  2. Both  3. None  4. others family members……………….. |
| 17 | | Father Monthly income | NRS………………….. |
| 18 | | Mother Monthly Income | NRS………………… |
| 19. | | Monthly Family income | NRS……………………… |

**Section B: Knowledge about Cervical cancer**

| 1 | Have you ever heard of cervical cancer? | 0. No  1. Yes | **If No (Go to section c)** |
| --- | --- | --- | --- |
| 2 | Where did you get the information about cervical cancer from?  **(Multiple choice)** | 1. Newspapers 2. Radio /Television 3. Internet 4. Healthcare providers 5. School 6. Friends and family 7. Parents 8. Other specify………….. |  |
| 3 | Cervical cancer is the most common female cancer? | 0. Yes  1. No  2. I don’t know |  |
| 4 | Organs affected by cervical cancer? | 0. Uterus  1. Breast  2. Cervix  3. I don’t know  4. Others…………………….. |  |
| 5 | Mode of acquisition/ transmission of cervical cancer  (Multiple Choice) | 1. Sexual transmission 2. Air 3. Water 4. Sin 5. Witches 6. History of cancer in a family 7. I don’t know 8. Others……………. |  |
| 6 | Which do you think are the risk factors for cervical cancer? (more than one answer possible) | 1. Multiple sexual partners 2. Multiple pregnancies 3. Early age sexual intercourse 4. Early marriage 5. Abortion 6. HPV infection 7. Low socio-economic status 8. Long term OCP use 9. Alcohol 10. Smoking 11. I don’t know 12. Others…………………. |  |
| 7 | What are the signs and symptoms of cervical cancer? (more than one answer possible) | 1. No symptoms in early stage 2. Vaginal bleeding after sexual intercourse 3. Post-menopausal bleeding 4. Dyspareunia 5. Increase vaginal discharge 6. Persistent lower back pain 7. I don’t know 8. Other symptoms……… |  |
| **Section C: Knowledge of HPV infection** | | | |
| 1 | Human papillomavirus (HPV) is a virus that causes a sexually transmitted infection, have you ever heard of it? | 1. No 2. Yes | **If NO go to section D** |
| 2 | Where did you get the information about HPV infection? (Multiple choice) | 1. Newspapers 2. Radio /Television 3. Internet 4. Healthcare providers 5. School 6. Friends and family 7. Parents 8. Other specify |  |
| 3 | Who can contact HPV infection? | 1. Only men 2. Only women 3. Men and women 4. I don’t know |  |
| 4 | Which of these diseases are caused by HPV infection? (more than one answer possible) | 1. Cervical cancer 2. Breast cancer 3. Oropharyngeal cancer 4. Anal cancer 5. Genital warts 6. I don’t know |  |
| 5 | Which one do you think the Ways of preventing HPV diseases (more than one answer possible) | 1. Practicing abstinence (No sex) 2. HPV Vaccination 3. By using Condoms 4. Regular screening 5. Cannot be prevented 6. I don’t know 7. Others……….. |  |
| 6 | What are risk factors for HPV infection? (more than one answer possible) | 1. High frequency of sex partner exchange 2. Genital –skin to skin contact 3. Body fluids(bloods) 4. I don’t know 5. Others specify…….. |  |
| **Section D: Knowledge of HPV vaccination** | | | |
| 1 | Have you ever heard about the Human Papilloma Virus (HPV) vaccine which is given against cervical cancer? | 1. No, I haven’t 2. Yes, I haven | **If no, go to section E Q 2 and Q3** |
| 2 | Where do you get the information about HPV vaccine from?  **(Multiple choice)** | 1. Newspapers 2. Radio /Television 3. Internet 4. Healthcare providers 5. School 6. friends and family 7. Parents 8. Other specify…… |  |
| 3 | The HPV vaccine is most effective in people who are not sexually active. | 1. No 2. Yes 3. I don’t know |  |
| 4 | Who should get the HPV vaccination in Nepal? | 1. Only Men 2. Only Women 3. Men and women 4. I don’t know |  |
| 5 | The HPV vaccine helps to prevent | 1. Prevent vaginal cancer 2. Prevent cervical cancer 3. Prevent breast cancer 4. I don’t know 5. Other specify |  |
| 6 | Recommended doses of HPV-Vaccine | 1. One dose 2. Two doses 3. I don’t know |  |
| 7 | The ideal time HPV Vaccine best recommended globally | 1. Less than 9-years 2. 9–14 years 3. Can be provided at any age 4. I don’t know |  |
| 8 | Is the HPV vaccine available in Nepal? | 1. No 2. Yes 3. Don’t know | **If no/ don’t know go to section E** |
| 9 | If yes, where is it available? | 1. Government health facility 2. Private health facility 3. Both government and private health facility 4. Others specify………… |  |
| 10 | What age of girls are getting HPV vaccine in Nepal? | 1. <9 years 2. 9-13 years 3. 14 years 4. Don’t know |  |
| 11 | Does it guarantee 100% protection from cervical cancer | 1. No 2. Yes 3. Don’t know |  |
| 12 | Do you know the cost of HPV vaccination by the government in Nepal? | 1. Not free 2. Free of cost 3. Don’t know |  |

| **Section E: Presence of promotion and sources of information about HPV vaccination, HPV and cervical cancer (If heard about HPV vaccine before filling all questions in this section.** | | | |
| --- | --- | --- | --- |
| 1 | Are there any initiatives or campaigns promoting HPV vaccination awareness in your local area? | 1. No 2. Yes |  |
| **2** | Have you been taught about sexually transmitted diseases in school? | 1. No 2. Yes |  |
| **3** | How comfortable do you feel discussing sexual health related topics with your teachers? ( If not heard about HPV vaccine before go to Q9) | 1. Uncomfortable 2. Somewhat comfortable 3. comfortable |  |
| 4 | Have you been taught about HPV or cervical cancer at school? | 1. No 2. Yes | **If No go to Q-6** |
| 5 | In which subject you were taught? | Specify………………… |  |
| 6 | Has your school provided any information about the HPV vaccine? | 1. No 2. Yes | **If no go to Q-8** |
| 7 | If YES, what information did you get? (Tick any/all that apply) | 1. Transmission of HPV 2. Diseases caused by HPV 3. Prevention of HPV 4. HPV vaccine 5. Cervical Cancer 6. Other………….. |  |
| 8 | Do health workers teach you about cervical cancer or HPV through community outreach and other means? | 1. No 2. Yes |  |
| 9 | Do you feel you need more information about HPV vaccination? | 1. No 2. Yes (If yes go to Q 10) | If No, go to section F |
| 10 | If yes, from whom do you prefer? (More than one answer is possible) | 1. Mass media 2. Health Workers 3. School 4. Social media 5. Friends 6. Family or parents 7. Others Specify |  |

| **Section F: Perception towards cervical cancer, HPV infection and HPV vaccination** | | | | | | |
| --- | --- | --- | --- | --- | --- | --- |
| s.n | Questions | Likert scale Range | | | | |
|  |  | Strongly disagree | Disagree | Neutral | Agree | Strongly  agree |
| **Perceived susceptibility** | | | | | | |
| 1 | I am at risk of contracting HPV | 1 | 2 | 3 | 4 | 5 |
| 2 | HPV infection could lead to serious health issues such as cancer. | 1 | 2 | 3 | 4 | 5 |
| 3 | Any woman can be a victim of cervical cancer | 1 | 2 | 3 | 4 | 5 |
| 4 | I am concerned about contracting HPV. | 1 | 2 | 3 | 4 | 5 |
| **Severity** | | | | | | |
| 5 | HPV-related diseases are severe. | 1 | 2 | 3 | 4 | 5 |
| 6 | I am worried about the consequences of HPV infection. | 1 | 2 | 3 | 4 | 5 |
| 7 | HPV infection can have long-term effects on my health | 1 | 2 | 3 | 4 | 5 |

| **Perceived barriers (Barriers for HPV vaccine)** | | | | | | |
| --- | --- | --- | --- | --- | --- | --- |
| 8 | I am concerned about possible side effects | 1 | 2 | 3 | 4 | 5 |
| 9 | I am concerned about safety and effectiveness | 1 | 2 | 3 | 4 | 5 |
| 10 | I am concerned about cost of vaccination | 1 | 2 | 3 | 4 | 5 |
| 11 | My parents won’t allow me to take HPV vaccination | 1 | 2 | 3 | 4 | 5 |
| 12 | Fear of needles or injections prevents me from getting vaccinated | 1 | 2 | 3 | 4 | 5 |
| 13 | I fear HPV vaccination due to cultural/religious reasons | 1 | 2 | 3 | 4 | 5 |
| 14 | I don't have access to information on HPV | 1 | 2 | 3 | 4 | 5 |
| 15 | I don't have access to vaccination | 1 | 2 | 3 | 4 | 5 |
| 16 | HPV vaccination will make me sexually unfit | 1 | 2 | 3 | 4 | 5 |
| **Perceived Benefits/facilitators (Facilitators for HPV vaccine)** | | | | | | |
| 17 | HPV vaccine is effective at preventing cervical cancer | 1 | 2 | 3 | 4 | 5 |
| 18 | HPV vaccination saves lives and improves health outcomes | 1 | 2 | 3 | 4 | 5 |
| 19 | Authoritative reassurances about safety and efficacy are given | 1 | 2 | 3 | 4 | 5 |
| 20 | HPV vaccine is recommended by doctors | 1 | 2 | 3 | 4 | 5 |
| 21 | My friends motivate me for HPV vaccination in school | 1 | 2 | 3 | 4 | 5 |
| 22 | HPV vaccine is recommended by the government | 1 | 2 | 3 | 4 | 5 |
| **Self-Efficacy** | | | | | | |
| 23 | I will receive HPV vaccination in future | 1 | 2 | 3 | 4 | 5 |
| 24 | I will be able to convince a hesitant family member or friend to get vaccinated against HPV | 1 | 2 | 3 | 4 | 5 |
| 25 | I will communicate effectively with healthcare providers about HPV vaccination | 1 | 2 | 3 | 4 | 5 |
| 26 | I will adhere to the recommended schedule for receiving multiple doses of HPV vaccine | 1 | 2 | 3 | 4 | 5 |
| 27 | HPV vaccination is important, and I will recommend it to others | 1 | 2 | 3 | 4 | 5 |
| 28 | I will advocate for improved access to HPV vaccination services in my community | 1 | 2 | 3 | 4 | 5 |
| **Cues to action** | | | | | | |
| 29 | Health care providers recommended me to get vaccinated | 1 | 2 | 3 | 4 | 5 |
| 30 | School/Health facilities/Community launched educational campaigns about HPV vaccination | 1 | 2 | 3 | 4 | 5 |

| **Section G: HPV vaccine uptake related information** | | | |
| --- | --- | --- | --- |
| 1 | Have you received HPV vaccination? | 1. No, I haven’t 2. Yes ,I have | If No, skip to Q-12 |
| 2 | If yes, how many doses of the vaccine have you received? | 1. One   1. Two |  |
| 3 | Mode of reporting | 0. Self-reported  1. vaccine card |  |
| 4 | Distance of house from vaccine delivery site (walking/vehicle) | In minutes…….. |  |
| 5 | Age at the time of vaccination | Complected years……… |  |
| 6 | Reason for taking only 1 dose | Specify………… |  |
| 7 | From where you receive the vaccine. | 0. Government health facility  1. Private health facility |  |
| 8 | If a government health facility from where you receive a vaccine? | 0. School  1. Community building  2. Health facility  3. Others………… |  |
| 9 | If from a private health facility then how much money paid? | Rs……………. |  |
| 10 | Did health workers provide full information about HPV vaccines before vaccination? | 1. No 2. Yes 3. I don’t know |  |
| 11 | If you received a vaccine, what helped you to receive it? (Possibly more than one answer) | 1. Pre-information 2. Believes in its benefit 3. Encouragement from health workers 4. Parental influence 5. Peer influence 6. Available free of cost 7. Other, specify……………… |  |
| 12 | If you haven’t taken the HPV vaccine before, why? (skip if you received)  (Multiple choice) | 0. No information about the vaccine  1. Negative attitude toward the vaccine  2. Being absent on vaccination day  3. Fear of side effects  4. Fear of needle injection  5. Parental concern  6. Peer influence  7. No belief in its benefit  8. Lack of access  9.Socialpressures/rumors/misconceptions  10. Fear of becoming infertile  11.Other specify | Only for unvaccinated |
| 13 | Are you willing to receive HPV vaccination in the future? | 1. No **(If no go to Q14)** 2. Yes **(If yes go to Q 15)** |  |
| 14 | Reason for not willing to take the vaccination  **(Multiple choice)** | 1. Lack of information about vaccine 2. Not considering myself at risk 3. No need to take the vaccine at an earlier age 4. Fear of needles/injection 5. Fear of side effects 6. Religious reason 7. Unavailability of vaccine 8. Parent’s hesitation 9. Other |  |
| 15 | Where do you prefer to get your HPV Vaccination? (vaccination site) | 1. Private Hospital 2. Government Hospital 3. School 4. Clinic 5. Others…………… |  |
| 16 | What best can be done to improve the acceptability of HPV vaccination ser-vices? (Multiple choice) | 1. Health education 2. Health worker recommendation 3. Information from teachers 4. Mass media information 5. Other specify |  |
