# Supplementary material for: Human papillomavirus (HPV) vaccine uptake and its associated factors among adolescent girls in Kathmandu District, Nepal: A cross-sectional study
Source: PLOS Glob Public Health. 2026 Jan 30;6(1):e0005893. doi: 10.1371/journal.pgph.0005893 (PMC12857967; doi:10.1371/journal.pgph.0005893)
Supplement: Attachment — Submitted filename: Response to Reviewers.docx [file pgph.0005893.s004.docx]

**October 5, 2025**

Dear Reviewers and Editors

Thank you very much for your valuable comments . They were very helpful to improve this manuscript. I have incorporated the suggested changes into the manuscript to the best of my ability. Please find the response to the comments.

**Journal Requirements:**

1. Please amend your detailed Financial Disclosure statement. This is published with the article. It must therefore be completed in full sentences and contain the exact wording you wish to be published.

**Response:** This study received financial support from the GTA Foundation Nepal through the Graduate Research Grant 2024.

2. Please clarify all sources of funding (financial or material support) for your study. List the grants (with grant number) or organizations (with url) that supported your study, including funding received from your institution.

**Response:** This study was financially supported by the GTA Foundation Nepal through the *Graduate Research Grant 2024*. The GTA Foundation Nepal (<https://gtafoundation.org.np/>) provided financial support for data collection . No additional financial or material support was received from any other organization or institution.

3. State the initials, alongside each funding source, of each author to receive each grant.

**Response:** The funding was awarded to the principal investigator, **ST** (Shristi Thapa).

4. State what role the funders took in the study. If the funders had no role in your study, please state: “The funders had no role in study design, data collection and analysis, decision to publish, or preparation of the manuscript.”

**Response:** “The funders had no role in study design, data collection and analysis, decision to publish, or preparation of the manuscript.”

5. If any authors received a salary from any of your funders, please state which authors and which funders.

**Response:** None of the authors received a salary or any other form of personal payment from the GTA Foundation Nepal

6. Please send a completed 'Competing Interests' statement, including any COIs declared by your co-authors. If you have no competing interests to declare, please state "The authors have declared that no competing interests exist". Otherwise please declare all competing interests beginning with the statement "I have read the journal's policy and the authors of this manuscript have the following competing interests:"

**Response:** "The authors have declared that no competing interests exist".

7. In the online submission form, you indicated that Data is available upon request.

All PLOS journals now require all data underlying the findings described in their manuscript to be freely available to other researchers, either 
1. In a public repository, 
2. Within the manuscript itself, or 
3. Uploaded as supplementary information.

This policy applies to all data except where public deposition would breach compliance with the protocol approved by your research ethics board. If your data cannot be made publicly available for ethical or legal reasons (e.g., public availability would compromise patient privacy), please explain your reasons by return email and your exemption request will be escalated to the editor for approval. Your exemption request will be handled independently and will not hold up the peer review process, but will need to be resolved should your manuscript be accepted for publication. One of the Editorial team will then be in touch if there are any issues.

**Response:** All relevant data underlying the findings of this study are submitted as supplementary information files within this manuscript.

8. If the reviewer comments include a recommendation to cite specific previously published works, please review and evaluate these publications to determine whether they are relevant and should be cited. There is no requirement to cite these works unless the editor has indicated otherwise.

**Response:** The reviewers did not provide any specific recommendations to cite previously published works. Therefore, no additional citations were added based on reviewer suggestions.

**Reviewer #1:**

**Comment:** Lines 50-52 need to be corrected or reframed.

**Response:** Thank you for your comment. I will correct and reframed Line 50-52 in revised manuscript as follows: More than 95% of cervical cancer cases are caused by infection with the Human Papillomavirus (HPV), which is the most common sexually transmitted infection worldwide.
**Comment:** Lines 53-55 can be edited to a sentence

**Response:** Line 53-55 will be edited to single sentence in manuscript as : South-East Asia has the highest incidence and mortality rates of cervical cancer, with approximately 69,000 new cases and 38,000 deaths reported in 2020.

**Comment:** Lines 55-57 should be revised

**Response:** Line 55-57 will be revised as: In Nepal, cervical cancer is the most common cancer among women, with a crude incidence rate of 14.2 per 100,000 women and an estimated 2,244 new cases and 1,493 deaths occurring annually.

**Comment:** The in-text references should come before the full stop.

**Response:** Thank you for the suggestion. We have revised all in-text references so that the references now appear before the full stop throughout the manuscript

**Comment**: The author should be consistent with the use of respondent throughout the manuscript.

**Response:** Thank you for the comment. We have carefully reviewed the manuscript and ensured that the term **“respondents”** is used consistently throughout to refer to the study participants accordingly, wherever possible. All instances of alternative terms have been corrected.

Reviewer #2:

**Comment:** The manuscript reports the findings of a study that aims to understand the uptake of HPV vaccine among the school-going adolescent girls in Kathmandu district and the factors associated with this uptake. With various governments in the process of introducing this vaccine as part of their national immunization programmes, this study seems to contribute to the understanding of the factors associated with the uptake of the same.

The authors have described the methods and analysis approach in detail. Clarification on the ethical considerations especially because the study involves children are also made clear. Data is not made available but the authors have responded to the PLOS question on data availability that they would be shared upon request.

**Response:** We thank the reviewer for the comment. All data underlying the findings described in the manuscript will be added as **supplementary information file within the manuscript.**

**Comment**: One key consideration for the authors to further clarify in their manuscript is regarding the conceptualisation of vaccine uptake. To make it clear as to how is the process or phenomenon or issue of vaccine acceptance or uptake being viewed in this study? This will be useful for the authors to then substantiate the choice of the factors (or lack of) in the study. In the process of describing this, the authors then can explicitly mention the factors that couldn't be explored due to the nature of the conceptualization itself for eg: access to the vaccine, agency of the child and parents to name a few.

**Response:** Thank you for this insightful comment. In our study, HPV vaccine uptake was conceptualized as the proportion of those 14 to 15-year-old girls who had received two doses of the HPV vaccine within a six-month interval. We measured vaccine uptake using full vaccination status, which is defined as receiving two doses of the HPV vaccine within a six-month interval. Any adolescent who had received one or no dose of the HPV vaccine was considered not fully vaccinated. HPV vaccine uptake was determined using respondents' recall or vaccination cards. Adolescents were considered fully vaccinated if they self-reported to have received two doses of the HPV vaccine within a six-month interval. Which is under measurements section inside HPV vaccine uptake related information section of manuscript. This conceptualization guided the selection of factors included in the analysis, focusing primarily on individual- and school-level determinants, such as socio-demographic characteristics, knowledge, perception and availability of information.

We acknowledge that certain factors, including **vaccine access, parental decision-making, and broader health system influences**, could not be explored due to the cross-sectional study design and the focus on adolescent respondents. This clarification has been added and highlighted as the limitations of study as follows: certain factors that may influence HPV vaccine uptake: such as vaccine access, parental decision-making, and broader health system influences, were not assessed in this study due to its cross-sectional design and focus on adolescent respondents.
