# Supplementary material for: Human papillomavirus (HPV) vaccine uptake and its associated factors among adolescent girls in Kathmandu District, Nepal: A cross-sectional study
Source: PLOS Glob Public Health. 2026 Jan 30;6(1):e0005893. doi: 10.1371/journal.pgph.0005893 (PMC12857967; doi:10.1371/journal.pgph.0005893)
Supplement: Attachment — Submitted filename: Response to Reviewer.docx [file pgph.0005893.s005.docx]

Reviewer #3:

Thank you very much Reviewer 3 for your valuable comments . They were very helpful to improve this manuscript. I have incorporated the suggested changes into the manuscript to the best of my ability. Please find the response to the comments.

**Review Comments**
General Comments
**Comment:** The standard of the journal should be used consistently

**Response:** Thank you for the comment. We have reviewed the manuscript carefully and revised it to follow the journal’s standards consistently throughout manuscript.

**Comment:** The formatting needs change

**Response:** Thank you for the comment. The manuscript formatting has been revised according to the journal’s guidelines.
**Comment:** Use of present tense E.g. In line 89 this study aims?

**Response:** Thank you for the comment. The sentence has been reviewed and is already written in the present tense. We have retained it as: *“Therefore, this study aims to estimate the current prevalence of HPV vaccine uptake.”* to ensure consistency throughout the manuscript.
**Comment:** Sections fail to contain what it should scientifically contain.

**Response:** Thank you for the comment. All sections of the manuscript have been carefully reviewed and revised to ensure they scientifically contain the appropriate content. The Introduction now clearly presents the background, rationale, and objectives of the study; the Methods section includes detailed information on study design, setting, population, sampling, data collection, variables, and analysis; the Results section presents the findings clearly and systematically; and the Discussion interprets the results in the context of existing literature, addresses implications, strengths, and limitations of the study.
**Specific Comments
On the scope**
**comment:** Is that post HPV vaccination survey or not?

**Response**: Yes, the study was conducted after the HPV vaccination program had been implemented in the selected schools/areas; therefore, it is a post-HPV vaccination survey. We have clarified this point in the Methods section of the manuscript to avoid any ambiguity.
**Comment:** If Vaccination is given for them, why you intended to study to it?

**Response:** Although the HPV vaccine had already been provided in the selected areas, there is still a critical need to study the actual uptake, coverage, and associated factors. Vaccination programs do not guarantee that all eligible individuals receive the vaccine. This study was therefore intended to assess the current level of HPV vaccine uptake and to identify the factors influencing vaccination among adolescent girls.

**Comment:** After administration of the vaccination, could we study uptake or hesitancy?

**Response:** Thank you for the comment. In this study, we focused specifically on HPV vaccine uptake, which refers to whether the eligible adolescent girls actually received the vaccine. Although vaccine hesitancy is an important aspect, it was not within the scope of this study.

**Comment:** Do the eight municipalities part of the Kathmandu district?

**Response:** Yes, all eight municipalities included in the study are part of Kathmandu district. This has been clarified in the Methods section (lines 95–97) of the manuscript.

**On the Title**
**Comment:** Add year and possibly the study design to the title

**Response:** Thank you for the suggestion. We had revised the title ad addesed study design and country as follow**: “Human Papillomavirus (HPV) Vaccine Uptake and Its Associated Factors among Adolescent Girls in Kathmandu District, Nepal: A Cross-Sectional Study”
On the Abstract**
**Comment:** Incomplete objective E.g. Add Nepal after the district

**Response:** Thank you for the comment. We have included word Nepal and revised the objective as: This study aims to assess the prevalence of HPV vaccine uptake and its associated factors among adolescent girls in the Kathmandu district, Nepal.

**Comment:** In line 13-14 you stated that cervical cancer is the most common cancer among women.... How most common is that?

**Response: Thank you for the comment. We have revised line as follows in the manuscript :** In Nepal, cervical cancer is the second most common cancer with crude incidence of 14.2 per 100000 women.

**Comment:** The objective can be merged to the background and be part of the background at the end of the paragraph.

**Response:** Thank you for the suggestion. We have now merged the study objective into the background section and placed it at the end of paragraph in the manuscript
**Comment:** In line 22 The participants completed a self-administered survey. Needs rewriting.

**Response:** We have rewrite the line as follows: Participants completed a supervised self-administered questionnaire.

**Comment:** The methods section is incomplete.

**Response:** Thank you for pointing out that the Methods section required more completeness. We have now expanded and clarified the section to provide sufficient detail on the study design, sampling procedure, data collection process, outcome measurement, and statistical analysis. Now the method section read as follows**:** We conducted a cross-sectional study in Kathmandu district, Nepal, from July to September 2024. Using multistage cluster random sampling, 142 schools were selected from 770 across eight municipalities where HPV vaccination was available. Ten girls from grades 9 and 10 were randomly selected per school, enrolling 1,510 adolescents aged 14–15 years. Participants completed a supervised self-administered questionnaire. HPV vaccine uptake was defined as receipt of two doses within six months. Factors associated with uptake were analyzed using Poisson regression with generalized estimating equations, accounting for clustering and sampleweights.
**Comment:** The prevalence among 14-15-year-old girls were 12.74%. is doubtable. Where are the other adolescents? If they enter to school at age 7 their current age at grade nine and ten will be 16 and 17. Therefore what are you saying?

**Response:** Thank you for raising this concern. In the context of Nepal, the age range of students in grades 9 and 10 can vary because many children start school earlier than age 5. As a result, it is common to find adolescents aged 14–15 as well as older adolescents in these grades. For this study, we restricted eligibility to girls aged 14–15 years because this age group aligns with the recommended age for HPV vaccination and with the focus of our research objectives. Therefore, the reported prevalence of 12.74% specifically reflects vaccine uptake among the enrolled 14–15-year-old girls who met the inclusion criteria.

**Comment:** The result should contain Confidence interval for the prevalence.

**Response:** Thank you for the comment. We have now added the confidence interval for the prevalence in result. The revised text reads: *“HPV vaccine uptake among 14–15-year-old girls was 12.74% ( CI: 7.2–21.4).”*

**Comment:** The result is inconsistent E.g. Why you use the APR, CI and the P value interchangeably.

**Response: We appreciate the comment. The manuscript has been revised to report all results consistently using Adjusted Prevalence Ratios (APR) with Confidence Interval as follows:** The prevalence of HPV vaccine uptake was 12.74% (CI: 7.2–21.4). Girls attending private schools had significantly lower uptake compared with those in public schools (APR: 0.05; CI: 0.02–0.13), while 15-year-old girls had higher uptake than 14-year-olds (APR: 2.28; CI: 1.49–3.50). Lower uptake was observed among girls whose fathers were daily wage laborers (APR: 0.59; CI: 0.39–0.90), self-employed (APR: 0.59; CI: 0.39–0.90), or employed abroad (APR: 0.65; CI: 0.43–0.97) compared with those whose fathers worked in agriculture. Conversely, higher uptake was noted among girls whose mothers were employed in government jobs (APR: 2.70; CI: 1.55–4.69), private jobs (APR: 2.15; CI: 1.11–4.14), or abroad (APR: 2.58; CI: 1.31–3.86). Good knowledge of HPV infection (APR: 1.88; CI: 1.13–3.15) and good or moderate knowledge of the HPV vaccine (APR: 2.73; CI: 1.33–5.60 and APR: 2.02; CI: 1.07–3.82, respectively) were also significantly associated with higher uptake.

**Comment:** Inconsistency in use of words E.g. 1.88 times,45% lower, more likely

**Response:** Thank you for the comment. We have revised the manuscript to ensure consistency in reporting effect estimates. All results are now uniformly expressed as Adjusted Prevalence Ratios (APR) with Confidence Intervals, avoiding mixed terms such as “times,” “lower,” or “more likely” as follows: The prevalence of HPV vaccine uptake was 12.74% (CI: 7.2–21.4). Girls attending private schools had significantly lower uptake compared with those in public schools (APR: 0.05; CI: 0.02–0.13), while 15-year-old girls had higher uptake than 14-year-olds (APR: 2.28; CI: 1.49–3.50). Lower uptake was observed among girls whose fathers were daily wage laborers (APR: 0.59; CI: 0.39–0.90), self-employed (APR: 0.59; CI: 0.39–0.90), or employed abroad (APR: 0.65; CI: 0.43–0.97) compared with those whose fathers worked in agriculture. Conversely, higher uptake was noted among girls whose mothers were employed in government jobs (APR: 2.70; CI: 1.55–4.69), private jobs (APR: 2.15; CI: 1.11–4.14), or abroad (APR: 2.58; CI: 1.31–3.86). Good knowledge of HPV infection (APR: 1.88; CI: 1.13–3.15) and good or moderate knowledge of the HPV vaccine (APR: 2.73; CI: 1.33–5.60 and APR: 2.02; CI: 1.07–3.82, respectively) were also significantly associated with higher uptake.

**Comment:** The conclusion is weak and it lacks recommendation.

**Response:** Thank you for the comment. We have strengthened the Conclusion by clearly summarizing the main findings and adding actionable recommendations based on the results as follows: This study found that only 12.7% of girls aged 14–15 years in Kathmandu had received the HPV vaccine. Uptake was higher among public school students, older adolescents, and those with working mothers or good HPV knowledge. These findings underscore the need for targeted interventions to raise awareness, strengthen school-based health education, and improve access to vaccination services to enhance HPV vaccine coverage.

**Comment:** The key words are incomplete

**Response:** Thank you for the comment. We have revised the keywords to make them more comprehensive and reflective of the study. The updated keywords are: HPV vaccine, HPV infection, cervical cancer, vaccine uptake, adolescent girls, school-based vaccination, knowledge, Nepal, Kathmandu district.

**Introduction**
**Comment:** Regional magnitude is missing

**Response:** Thank you for the comment. We have now included information on the regional magnitude of cervical cancer in the Introduction section first paragraph as follows: South-East Asia has the highest incidence and mortality rates of cervical cancer[3], with approximately 69,000 new cases and 38,000 deaths reported in 2020 [4]. In Nepal, cervical cancer is the most common cancer among women, with a crude incidence rate of 14.2 per 100,000 women and an estimated 2,244 new cases and 1,493 deaths occurring annually[5]. Cervical cancer ranks as the first leading cause of female cancer and the second most common cancer in women aged 15 to 44 years in Nepal [6].

**Comment:** The definition, causative agent, severity, associated factors and efforts made globally and regionally as well as nationally were missed.

**Response:** Thank you for the comment. We have revised the Introduction to provide a comprehensive background that includes the definition of cervical cancer, its causative agent (HPV), the severity and burden of the disease globally, regionally, and nationally. Now the revised introduction reads as follows: “Cervical cancer defined as a malignant tumor of the cervix primarily caused by persistent infection with the Human Papillomavirus (HPV) is a major public health problem globally ranking as the fourth leading cause of cancer death among women, with 662,301 new cases and 348,874 deaths of women in 2022 [1]. More than 95% of cervical cancer cases are caused by infection with the Human Papillomavirus (HPV), which is the most common sexually transmitted infection worldwide[2]. About 90% of deaths caused by cervical cancer occurred in low and middle-income countries (LMIC)[3]. South-East Asia has the highest incidence and mortality rates of cervical cancer[3], with approximately 69,000 new cases and 38,000 deaths reported in 2020 [4]. In Nepal, cervical cancer is the most common cancer among women, with a crude incidence rate of 14.2 per 100,000 women and an estimated 2,244 new cases and 1,493 deaths occurring annually[5]. Cervical cancer ranks as the first leading cause of female cancer and the second most common cancer in women aged 15 to 44 years in Nepal [6].

The World Health Organization recommends two doses of the HPV vaccine for 9 to 14-year-old girls, which will act as a primary prevention against cervical cancer if it is administered before being sexually active [7]. HPV immunization could control nearly 70% of all cervical cancers [8,9] . HPV vaccines are available in at least 124 countries, including Nepal[10]. Sixty-four countries have launched national HPV immunization programs to achieve the 2030 Sustainable Development Goal (SDG) of vaccinating 90% of girls by age 15 [11]. However, global coverage with the first dose of HPV among girls is now estimated at 21%.[3] Nepal does not have a national HPV immunization program, and no national-level vaccination coverage data is available[7].

In Nepal, there was a pilot project to vaccinate (11- 13) year-old girls in two districts from 2016 to 2017 with two doses of Cervarix. However, escalating it and incorporating the program into the regular vaccination program was not achieved [7]. In 2018, a study conducted among adolescent girls in Nepal revealed that only 13.9% of school-going girls were aware of the HPV vaccine. In 2023, the Ministry of Health and Population launched a broader HPV vaccination campaign, purchasing 20,000 HPV vaccines and distributing them through seven major hospitals nationwide. The campaign targeted 14-year-old school-going adolescent girls, administering two vaccine doses over a six-month period [12,13]. But the vaccine coverage is low. For example, only around 600 girls in Kathmandu district have been inoculated with the First dose of the HPV vaccine, although health authorities have targeted to vaccinate 3000 girls [12]. The Family Welfare Division, Department of Health Services (DoHS), releases a new guideline on HPV Vaccine Demonstration 2024 to provide guidance on the HPV vaccine demonstration program in Nepal and a new guideline on HPV Vaccination Service Operational Guidelines 2024 to provide guidance, operational and procedures on the HPV Vaccination service program in Nepal [14]. Nepal's Ministry of Health and Population (MoHP) has announced plans to launch a nationwide HPV vaccination campaign on February 4, 2025. The campaign targets 10-14-year-old girls, regardless of their school enrollment status [15]. Despite these and future targets from the Nepali government, there is a limited understanding of the factors affecting vaccination uptake in Nepal’s context. Existing studies indicate low awareness and limited access to vaccination services, but evidence from Kathmandu district is scarce. Addressing this knowledge gap is critical to design effective interventions and policies to improve vaccination coverage. Factors must be identified earlier and addressed to run an effective vaccine program and increase vaccine uptake among adolescent girls. Therefore, this study aims to estimate the current prevalence of HPV vaccine uptake in the Kathmandu district and identify factors associated with vaccine uptake. “

**Comment:** The knowledge gap is widely missed

**Response:** Thank you for the comment. We acknowledge that the knowledge gap regarding HPV vaccine uptake was not clearly highlighted in the original Introduction so we added the knowledge gap as follow in the manuscript**: “**Despite these and future targets from the Nepali government, there is a limited understanding of the factors affecting vaccination uptake in Nepal’s context. Existing studies indicate low awareness and limited access to vaccination services, but evidence from Kathmandu district is scarce. Addressing this knowledge gap is critical to design effective interventions and policies to improve vaccination coverage. Factors must be identified earlier and addressed to run an effective vaccine program and increase vaccine uptake among adolescent girls. Therefore, this study aims to estimate the current prevalence of HPV vaccine uptake in the Kathmandu district and identify factors associated with vaccine uptake”.

**Methods
Comment:** The study setting is incomplete E.g. How many health facilities and what type of services are available in the setting?

**Response: Thank you for the comment. We did not include health facility information because this study was school-based and focused on adolescent girls’ HPV vaccine uptake. The study setting comprised selected schools in Kathmandu district where HPV vaccination services were available.** While HPV vaccination was available through health facilities, detailed information on the number or type of health facilities and services was not collected, as it was beyond the scope of the current study.
**Comment:** The study design is not written

**Response:** Thank you for your comment. The study design has been described in the manuscript in the section titled “Study Setting and Design” under line 101, where we state that: This is a cross-sectional study among adolescent school girls aged 14 to 15 years in eight municipalities of Kathmandu District.

**Comment:** The source and the study population were not correctly written

**Response:** Thank you for the comment. We have revised the manuscript to clearly and accurately distinguish between the source and the study population. The revised text in the manuscript now reads as follows: “*The study population comprised school-going adolescent girls aged 14–15 years who were enrolled in grades 9 and 10 in all eligible secondary-level schools across eight municipalities of Kathmandu district during the study period, as identified from official school lists obtained from the respective municipalities”.*

**Comment:** The inclusion criteria are not firm.

**Response:** Thank you for the comment. We have clarified and strengthened the inclusion criteria in the manuscript. The inclusion criteria are clearly defined as: school-going adolescent girls aged 14–15 years who were enrolled in eligible schools within the study area at the time of data collection, in alignment with the Ministry of Health and Population (MOHP) 2023 HPV vaccination initiative targeting school-going 14-year-old girls.

**Comment:** Is cluster appropriate sampling procedure for this study?

**Response:** Thank you for the comment. Yes, cluster sampling was an appropriate and justified sampling procedure for this study. Schools naturally served as clusters because the target population school-going adolescent girls aged 14–15 years was organized within schools. Given the large geographic coverage and the absence of an individual-level sampling frame, cluster sampling was both logistically feasible and methodologically suitable. This approach also minimized disruption to school activities and reduced time and resource constraints while ensuring adequate representation across the selected municipalities**.**
**Comment:** If you have sampling frame why you used systematic sampling?

**Response:** Thank you for the comment. Although a sampling frame of eligible adolescent girls was available at the school level, systematic sampling was used to ensure a simple, transparent, and unbiased selection of participants within each selected school. After listing all eligible girls according to class roll numbers, a sampling interval was calculated by dividing the total number of eligible girls by the required sample size per school. A random starting point was then selected, and every *nth* student was chosen until the desired number was reached. This method was appropriate for within-cluster participant selection and helped ensure equal probability of selection while maintaining feasibility in the school setting.
**Comment:** What is the source of the tool, why used structured and what does that mean? What was the duration of the question [time frame]?

**Response**: Thank you for the comment. The data collection tool was developed based on an extensive review of relevant literature and questionnaires used in similar studies, and was adapted to the local context. A structured questionnaire was used to ensure uniformity and consistency in data collection across all participants; this means that all respondents were asked the same set of pre-defined questions in the same order, which facilitated comparability and quantitative analysis. The questionnaire captured information referring to the participants’ current status and recent experiences, with a recall period aligned to the time of data collection (i.e., information up to the survey date), and the overall duration required to complete the questionnaire was approximately 30 minutes**.**
**Comment:** Rather than pre-test, what was done to ensure quality? What type of validity and reliability?

**Response:** Thank you for the comment. Multiple measures were undertaken to ensure data quality beyond pretesting and all this information was stated within the manuscript under data quality section as follows: The structured questionnaire was initially developed in English, translated into Nepali, and then back-translated into English to ensure linguistic accuracy and consistency. Content validity was established through expert review by public health and subject-matter experts. To further ensure quality, data collectors were trained on the study objectives, ethical considerations, and standardized data collection procedures prior to fieldwork. The tool was pretested on 10% of the total sample size in a similar setting in Bhaktapur district to assess clarity, flow, feasibility, and consistency of responses; based on the findings, minor revisions were made to improve the questionnaire. Reliability was assessed using internal consistency analysis (Cronbach’s alpha). The alpha values were 0.72 for knowledge about cervical cancer, 0.98 for knowledge about HPV infection, 0.84 for knowledge about the HPV vaccine, and 0.84 for perception related to cervical cancer, HPV infection, and the HPV vaccine. The overall Cronbach’s alpha for the tool was 0.87, indicating good reliability. Additionally, the principal investigator and supervisor conducted regular supervision, spot checks, and reviews of completed questionnaires to ensure completeness and consistency. Data were entered daily into the Kobo Collect application by the principal investigator to minimize entry errors and maintain data accuracy**.**

**Top of Form**

**Bottom of Form**

**Comment:** The ethics is incomplete

**Response**: Thank you for the comment. A complete ethical statement was included within the manuscript in Ethical Statement section as follows: “The ethical clearance was obtained from the Institutional Review Committee (IRC) of Kathmandu University School of Medical Sciences (IRC-KUSMS Approval NO. 68/24). The study team obtained written informed consent from the respondents' parents and assent from the respondents. Respondents were informed about the research details, including objectives, their role in the study, and the risks and benefits of participation. Participation was voluntary, and withdrawal from the study at any time during data collection was considered. No name or other identifying information was included in the questionnaire.”

**Results**
Comments: It should be written well. E.g. For instance when do we say most?

Response: Thank you for the comment. We have revised the manuscript to ensure clarity and precision in reporting. Terms such as “most,” “majority,” “few,” or “some” are now used consistently based on the proportion of respondents
Comment: Tables are not self-explanatory E.g. Table 2.

Response: Thank you for the comment. We have revised all tables, including Table 2, to make them self-explanatory. Now the table 2 reas as follows in manuscript:

| **Level of knowledge** | **Weighted percent%** | **Weighted frequency** | **Unweighted frequency** |
| --- | --- | --- | --- |
| **Knowledge about Cervical cancer** | | | |
| Poor knowledge | 57.38 | 866 | 960 |
| Moderate Knowledge | 19.57 | 295 | 191 |
| Good knowledge | 23.05 | 384 | 359 |
| Mean (SD) |  | 33.28 (39.51) | 30.03(40.3) |
| **Knowledge about HPV infection** | | | |
| Poor knowledge | 79.72 | 1204 | 1227 |
| Moderate knowledge | 3.08 | 46 | 29 |
| Good knowledge | 17.20 | 260 | 254 |
| Mean (SD) |  | 18.53(35.87) | 17.44(35.76) |
| **Knowledge about HPV vaccine** | | | |
| Poor knowledge | 79.36 | 1198 | 1269 |
| Moderate Knowledge | 13.13 | 198 | 144 |
| Good Knowledge | 7.51 | 113 | 97 |
| Mean (SD) |  | 17.73(31.79) | 13.70(29.36) |
| **Perception towards cervical cancer, HPV infection, and HPV vaccination** | | | |
| Negative Perception | 45.49 | 687 | 733 |
| Positive perception | 54.51 | 823 | 777 |
| Mean (SD) |  | 101.49 (14.164) | 100.60(12.93) |

**Comment:** The results section can be widen more than this. Check for the statistics?

**Response:** Thank you for the comment. We appreciate the suggestion; however, the results section has been presented concisely to focus on key findings relevant to the study objectives. Expanding further may include redundant details and potentially obscure the main outcomes. All reported statistics have been carefully checked and accurately reflect the data analysis.
**Comment:** Differentiate which variables are associated in binary and multiple logistic Poisson regression?

**Response:** Thank you for the comment. We have differentiated the variables associated with the bivariate (binary) and multivariable Poisson regression analyses and have also included the interpretation of the bivariate results section as follows: Table 3 shows the factors associated with HPV vaccine uptake. In the bivariate model, respondents studying in private schools had a 96% lower prevalence of receiving the HPV vaccine compared to those in public schools (CPR: 0.04; 95% CI: 0.01–0.12; p < 0.0001). Respondents aged 15 had 95% higher prevalence of receiving the HPV vaccine compared to those aged 14 years (CPR: 1.95; 95% CI: 1.23–3.08; p = 0.003). Respondents whose fathers were self-employed in business had a 26% lower prevalence of receiving the HPV vaccine compared to those whose fathers worked in agriculture (CPR: 0.74; 95% CI: 0.56–0.99; p = 0.04). Respondents whose mothers held government jobs had a 53% higher prevalence of receiving the HPV vaccine compared to those whose mothers were homemakers (CPR: 1.53; 95% CI: 1.05–2.25; p = 0.02). A higher household annual per capita income significantly lowered the HPV vaccine prevalence (CPR: 0.99; 95% CI: 0.99–0.99; p = 0.01).

Table 4 presents the association between HPV vaccine uptake and respondents’ knowledge and perception levels. In the bivariate model respondents with good knowledge of cervical cancer exhibited a 2.03 times higher prevalence of HPV vaccine uptake compared to those with poor knowledge (CPR: 2.03; 95% CI: 1.24–3.34; p = 0.005)., adolescent girls with good knowledge of HPV infection had a 2.72 times higher prevalence of receiving the HPV vaccine compared to those with poor knowledge (CPR: 2.72; 95% CI: 1.51–4.88; p = 0.001). Participants with good knowledge of the HPV vaccine had a 3.91 times higher prevalence of receiving the vaccine (CPR: 3.91; 95% CI: 1.51–10.11; p = 0.005), while those with moderate knowledge showed a 2.73 times higher prevalence (CPR: 2.73; 95% CI: 1.31–5.71; p = 0.007) compared to those with poor knowledge.

**Discussion and the consequent sections
Comment:** The discussion is weak.

**Response:** Thank you for this comment. We have substantially strengthened the Discussion section by rewriting it to provide clearer, more coherent explanations supported by relevant literature. The revised section now integrates both theoretical perspectives and practical implications, and appropriate references have been added to strengthen the interpretation of findings and improve the overall scientific rigor. Below is the revised discussion: “ In this study, only about 12.7% of adolescent girls in Kathmandu District had received the HPV vaccine, indicating that uptake remains quite low. Most of the respondents did not know much about cervical cancer (57%), HPV infection (80%), or the HPV vaccine (79%). Approximately 46% of respondents had a negative perception about the vaccine. However, (79%) of respondents who had not been vaccinated said they were willing to get the vaccine. Several factors were linked to HPV vaccination. Respondents who were 15 years old, attended public schools, came from higher-income families, or had parents with formal jobs were more likely to be vaccinated. Those who had better knowledge about HPV and the vaccine were also more likely to have received it.

Our study found that HPV vaccination uptake among adolescent girls in Kathmandu Valley was 12.7%, lower than the 90% coverage reported during pilot vaccination in Nepal’s Chitwan and Kaski districts [7]. The pilot achieved high coverage which might be due to its controlled setting, close monitoring, and targeted delivery to over 14000 girls aged 11 and 13, and 10-year-olds out of school at health facilities [7]. Compared to neighbouring and high-income countries, HPV vaccine uptake in our study remains substantially lower as the prevalence of HPV vaccine uptake is lower than the study conducted in India (74.4%), Uttar Pradesh (UP) (79.17%), China (66.9%)[22–24], Taiwan (91%) and in Scotland (94.4%) [25]. These countries benefit from well-established healthcare systems and national immunization programs that support broader vaccine access [22–24]. In Nepal, the government’s 2023 demonstration campaign was limited in scope, targeting only 14-year-old schoolgirls with 20,000 doses at the time of the study. In contrast, Nepal’s uptake closely resembles rates reported in Uganda (8.6%) [20] and Ibadan, Nigeria (4.1%) [26]. Across these settings, studies consistently identify limited awareness, misinformation, cultural stigma surrounding adolescent reproductive health, and inconsistent vaccine supply as key barriers to vaccination [20,26]. Additionally, lack of information regarding vaccine availability emerged as a common reason for non-vaccination, aligning with findings from Ethiopia and Uganda [20].

We found that adolescent girls attending public schools and those aged 15 years were significantly more likely to be vaccinated than their counterparts. This pattern reflects Nepal’s campaign eligibility criteria, which primarily targeted 14-year-old girls enrolled in public schools, thereby excluding private school students and those outside the specified age range [12,13]. These findings underscore how policy design and implementation strategies directly shape vaccine uptake and may inadvertently create inequities.

Parental occupation also played an important role. Respondents whose fathers were daily wage workers, self-employed, or employed abroad had 41%, 35%, and 39% lower prevalence of HPV vaccine uptake, respectively, compared to those whose fathers worked in agriculture. This contrasts with studies from Ethiopia and Uganda, where fathers' occupation was not significantly associated with vaccine uptake among adolescent girls [20,27]. This may be because fathers engaged in daily wage work, self-employment, or foreign employment often face barriers such as irregular work hours, limited access to health information, and reduced involvement in their children’s health decisions due to demanding work schedules. In contrast, adolescent girls whose mothers held government jobs were 2.7 times more likely to receive the HPV vaccine, those with mothers in private jobs were 2.15 times more likely, and those whose mothers worked abroad were 2.58 times more likely, compared to girls whose mothers were homemakers. These findings align with a study in Ethiopia, which also found a significant association between mothers' occupation and HPV vaccine uptake [18]. A study conducted in Kampala, Uganda, found that adolescent girls whose mothers worked in healthcare had a 1.94 times higher likelihood of being fully vaccinated against HPV [20]. Similarly, in Nepal, mothers employed in government or private sectors or working abroad may have better access to health information, greater exposure to health campaigns, and more financial autonomy, enabling them to make informed decisions about their children’s health.

We also observed 45% lower HPV vaccine uptake among girls from households where mothers were the sole earners compared to those where fathers were the sole earners. This finding differs from studies in Uganda and India, which reported no significant association between household income source and vaccine uptake[18,22]. In Nepal, patriarchal norms often grant male earners more decision-making power over healthcare. Sole-earning mothers may face challenges balancing work and caregiving, limiting their ability to prioritize preventive health. Additionally, stigma and financial instability in women-headed households may further restrict access to vaccination services [7].

In our study, religion, ethnicity, and parental education were not significantly associated with HPV vaccine uptake, unlike findings from India, the U.S, and Malaysia [28,29]. This may be due to Nepal’s 2023 HPV vaccination campaign, which targeted public schools uniformly, minimizing disparities in access across religious and ethnic groups[12,13]. Moreover, the campaign’s school-based approach likely reduced the influence of parental education by ensuring equal access to information and vaccination for all eligible students.

Knowledge-related factors played a critical role in vaccine uptake. In our study, knowledge of cervical cancer was associated with HPV vaccine uptake in the bivariate analysis but not in the multivariate model. This contrasts with findings from Ethiopia, where girls with good cervical cancer knowledge were 2–2.8 times more likely to be vaccinated [30,31]. In Nepal, awareness of cervical cancer may not directly lead to vaccination if individuals are unaware that the HPV vaccine prevents it. Our findings showed vaccine uptake was more strongly linked to knowledge, specifically about the HPV vaccine. Structural barriers such as limited information, vaccine availability, cost, and access, along with parental influence on health decisions, may further limit uptake despite awareness [5].

Adolescent girls with good knowledge of HPV infection were twice as likely to receive the vaccine, while those with good knowledge of the HPV vaccine had the strongest association—nearly four times higher uptake in crude analysis and 2.74 times higher after adjustment. These findings align with studies from Ethiopia and Tanzania, where vaccine uptake was four times higher among knowledgeable girls [32,33]. Awareness of HPV and its vaccine enhances understanding of risks, dispels myths, and fosters trust in vaccination, leading adolescents to seek information, make informed choices, and influence parental decisions—ultimately improving uptake [32,33]. In both bivariate and multivariate models, no association was found between perceptions of cervical cancer, HPV infection, or HPV vaccination and vaccine uptake, contrasting with findings from Uganda where positive perceptions significantly increased uptake [20]. In Nepal, this lack of association may stem from barriers beyond perception, such as limited vaccine access, cultural norms, and socio-economic constraints. Moreover, adolescent vaccination decisions often depend on parents or guardians, whose views may differ from the adolescents’ own perceptions [7].

To our knowledge, this is the first study in Nepal to examine HPV vaccine uptake and associated factors among adolescent girls. With a large sample of 1,510 girls aged 14–15, the findings are robust and generalizable within similar settings. We used Generalized Estimating Equations to account for clustering by school and applied sample weights, with prevalence ratios providing clear and accurate associations. However, the study has limitations. It was conducted solely in urban Kathmandu, excluding rural areas and out-of-school girls, which may affect broader applicability. Additionally, the focus on 14–15-year-olds limits generalization to the recommended age range of 10–19 years. Finally, certain factors that may influence HPV vaccine uptake: such as vaccine access, parental decision-making, and broader health system influences, were not assessed in this study due to its cross-sectional design and focus on adolescent respondents.”

**Comment:** Some of the explanations lack reference E.g. In line 335-39These low rates 335 across Nepal, Uganda, and Nigeria reflect shared challenges, including limited awareness, misinformation, cultural stigma surrounding reproductive health, and logistical issues such as inconsistent vaccine supply. Many cited a lack of information about vaccine availability as a key reason for not getting vaccinated, findings that align with studies from Ethiopia and Uganda” It should entail appropriate explanation with necessary references employing both theoretical and practical implications

**Response:** Thank you for this comment. We have revised lines 335–339 to provide a clearer explanation supported by relevant literature and to integrate both theoretical and practical implications and added reference aswell.
**Comment:** Avoid some of the sentences E.g. The focus of the government of Nepal on adolescents aged 14 and 15 years [you can put it in the introduction section].

**Response:** Thank you for this comment. We have revised the manuscript by removing the sentence referring to the Government of Nepal’s focus on adolescents aged 14 and 15 years from the Results/Discussion section.
**Comment:** Why you acknowledge the department?

**Response:** We acknowledged the Department of Public Health, Kathmandu University School of Medical Sciences, in recognition of the academic and administrative support provided during the study, including guidance during study implementation and facilitation of the research process. We also acknowledged the data collectors and study participants for their valuable contributions.
**Comment:** What is the role of other authors?

**Response****:** Authors' contributions have been added to the manuscript as follows: ST and AS conceived the study. ST, AS, BP and PT contributed to the design of the study. ST, AS, PT, BP and AP contributed to development of data collection tools, data collection, analysis and data management. ST, AS, PT, BP, YA and AP wrote the manuscript. ST, AS, PT, BP, YA and AP significantly contributed to manuscript revisions. All authors contributed to refinement and approved the final manuscript.

**Comment:** Is there conflict of interest?

**Response:** The authors declared that there was no conflict of interest. And this is stated in the submission system during submission.

Regards,
